# Regulation of telomerase towards tumor therapy

**DOI:** 10.1186/s13578-023-01181-6

**Published:** 2023-12-18

**Authors:** Siyu Yan, Song Lin, Hongxin Qiu, Xining Wang, Yijun He, Chuanle Wang, Yan Huang

**Affiliations:** 1 MOE Key Laboratory of Gene Function and Regulation and Guangzhou Key Laboratory of Healthy Aging, School of Life Sciences, Sun Yat-sen University, Guangzhou, 510275 China; 2 Department of Oncology, Sun Yat-sen Memorial Hospital, Sun Yat-sen University, Guangzhou, 510275 China; 3 Lumiere Therapeutics Co., Ltd., Suzhou, 215000 China

**Keywords:** Telomerase, Tumor, Immunotherapy

## Abstract

Cancer is an aging-related disease, while aging plays an important role in the development process of tumor, thus the two are inextricably associated. Telomere attrition is one of the recognized hallmark events of senescence. Hence, targeting telomerase which could extends telomere sequences to treat tumors is widely favored. Cancer cells rely on high activity of telomerase to maintain a strong proliferative potential. By inhibiting the expression or protein function of telomerase, the growth of cancer cells can be significantly suppressed. In addition, the human immune system itself has a defense system against malignant tumors. However, excessive cell division results in dramatic shortening on telomeres and decline in the function of immune organs that facilitates cancer cell evasion. It has been shown that increasing telomerase activity or telomere length of these immune cells can attenuate senescence, improve cellular viability, and enhance the immunosuppressive microenvironment of tumor. In this paper, we review the telomerase-targeting progress using different anti-tumor strategies from the perspectives of cancer cells and immune cells, respectively, as well as tracking the preclinical and clinical studies of some representative drugs for the prevention or treatment of tumors.

## Background

Replicative senescence is an irreversible state of cell growth arrest, also known as “Hayflick Limit” [1], which is caused by multiple mechanisms including telomere attrition, DNA damage response activation and epigenetic modifications [2]. The pathological development of cancer is highly associated with aging process, therefore several clinical therapeutics for malignant tumors are based on triggering senescence to inhibit growth and expansion of cancer cells [3]. Also in recent years, immunotherapy as CAR-T has been favored by scientists and clinical experts, and is considered as the fourth pillar of oncology treatment [4]. Effective immune system could kill cancer cells to protect organs from tumorigenesis. Age-related decline in immunity (also termed immunosenescence) has been linked to increase cancer risk in patients [5].

Human telomeres consist TTAGGG repeats and shelterin complex with dynamic structures at the ends of chromosomes, which can maintain genomic stability and integrity [6, 7]. Since the end replication problem, telomeres would be shortened like a mitotic clock during cell cycle process [8]. As a key hallmark of aging, telomere attrition is recognized to lead in DNA damage response activation and limitation of cell proliferation capacity [9]. However, telomeres could be elongated by telomerase in stem cells and cancer cells to maintain robust proliferation capacity. Human telomerase contains catalytic telomerase reverse transcriptase (TERT) and telomerase RNA subunit (TR) as the template for telomere replication [10]. Over 80% tumors extend telomeres depending on telomerase [11]. Hence, human telomerase activity or telomerase reverse transcriptase (TERT) is regarded as a diagnostic and therapeutic biomarker in cancer cells [12].

Telomerase, a pivotal enzyme involved in cellular processes, is subject to intricate regulatory mechanisms. One of the primary steps is to control telomerase activity in the transcription level of the human telomerase reverse transcriptase (hTERT) gene. Several transcription factors, such as c-Myc, hypoxia-inducible factor (HIF-1), Vitamin D receptor (VDR), and Sp1 et al., have been identified to bind to the core promoter region of hTERT, thereby modulating its transcriptional activity [13]. The hTERT gene spans an impressive 42 kilobases and comprises 16 exons. Notably, the mRNA derived from hTERT is subject to site-selective splicing, resulting in various spliced variants [14]. It is worth noting that, aside from the full-length transcripts, some spliced hTERT versions lack reverse transcriptase activity and, consequently, the capacity to extend telomeres [15]. Intriguingly, environmental factors have been revealed to influence the splicing pattern of hTERT, further emphasizing the complexity of its regulation [16].

The modulations of telomerase activity play fundamental roles in immunosenescence which is an essential phenotype during organism aging. Immune-related NF-κB pathway has been reported to involve in regulation of telomerase activity in leukemia cells [17] and inflammatory cells [18]. Antigen activated lymphocytes also express high-level telomerase to compensate for end loss problem after massive expansion [19]. TERT transcription would be temporarily rebooted in activated CD28^+^ T cells [20]. Moreover, several studies demonstrated short telomeres in peripheral blood mononuclear cells (PBMC) may relate to the risks of oncogenesis [21–23]. Therefore, modulating telomere length in immune cells has clinical implications for the treatment of hematologic or immune-related diseases.

Here we are summarizing telomere and telomerase regulation towards on cancer therapeutics mainly from three aspects. First one is based on the common inhibition of cellular telomerase activity to suppress tumors, and the second is to reverse or delay the immunosuppressive microenvironment of malignant tumors by targeting telomerase. Lastly, we propose several potential therapies that target non-canonical roles of telomerase.

### Telomerase inhibition in cancers

Telomere, as a replication clock of cell, limits the frequency of cell division, and most cancer cells would reactivate telomerase for maintaining telomere length to achieve replicative immortality. Early generations of TERC^−/−^ mouse seem to have epithelial cancer resistance, indicating that telomerase is associated with cancer development which depends on telomere length [24]. The tumor formation rate and survival rate in mouse with dual inactivation of TERC and INK4a were higher than those in the control group, and re-expression of TERC can complement this phenotype [25]. Many statistical data indicate that the activation of telomerase in cancer cells often symbolizes clinical malignancy in diagnosis, staging, and prognostic significance. A study on telomerase activity of breast cancer samples found that 92.5% of breast cancer lesions were telomerase positive, while almost no telomerase activity was detected in adjacent non-malignant lesions [26]. And the break fine needle aspiration samples from different patients also show that telomerase is related to malignant diseases: most of the samples with confirmed breast cancer have telomerase activity, while the patients who are underdiagnosis with telomerase positive samples will be diagnosed with cancer later [27]. This correlation exists in the diagnosis of lung cancer, pancreatic cancer, renal cancer and so on [28–30]. Knocking out TERC in mouse can induce cell cycle arrest and apoptosis of leukemia stem cells, while knocking down TERT in AML cells isolated from leukemia patients can gain similar results [31].

Therefore, inhibiting telomerase to induce replicative senescence and cell death is a feasible anticancer strategy. Compared with traditional chemotherapy drugs, telomerase inhibitors can theoretically target cancers in a specific manner. Based on the properties of telomerase, some designed inhibitors are identified and synthesized for cancer therapy research (Table 1).

Non-nucleosidic compound BIBR1532 is based on the structure of telomerase RNA binding domain (TRBD), which is a unique domain of hTERT protein. It can tightly bind FVYL motif on the thumb domain, selectively block the binding of the CR4/5 ring of hTR to hTERT, thereby inhibiting the activity of telomerase holoenzyme [32]. Many in vitro studies have demonstrated that BIBR1532 exhibits promising telomerase inhibitory ability in different cancer cells and it is expected to be developed as a universal anti-cancer drug [33–36]. However, BIBR1532 was also found to have strong cytotoxicity, which is consistent with the reported properties of the quinoline derivatives [37]. Nevertheless, BIBR1532 is still being accepted by researchers as an effective telomerase inhibitor to restrain the growth and progression of tumor cells. This inhibition can also be further enhanced by combining BIBR1532 with other tumor therapies, potentially providing improved treatment outcomes [38, 39]. Although no relevant clinical research data has been published, the preclinical study evaluated the effect of BIBR1532 in suppressing malignant tumor. In the model of feline oral squamous cell carcinoma, BIBR1532 can inhibit telomerase modulation and interfere with signal pathway of cell proliferation and survival, and exert a multi-level capabilities in anti-cancer [34]. However, telomerase highly expressed germ cell carcinoma is usually treated with cisplatin in clinical practice. Combined with BIBR1532 for long-term treatment (300PD), telomere shortening could be observed in cell models [40], but the related data has not yet been published in more complicated animal models. It also suggests that even telomerase inhibitors that perform well at the laboratory level may not be effective in vivo, possibly due to extensive reserved telomeres.

Imetelstat is also a relatively successful synthetic telomerase antagonist which is based on the lipid conjugation optimization of GRN163, thus also known as GRN163L [41]. This small molecule is characterized by 13 bases with the sequence 5’-TAGGGTTAGACAA-3’ that competitively binds to hTR [42]. Imetelstat has been found to delay G2/M checkpoint progression in telomerase positive cells by inhibiting telomerase activity [43]. Long-term Imetelstat treatment to cancer cells can accelerate telomere attrition and induce cellular senescence [44, 45]. Similar to BIBR1532, this small molecule can also be combined with other anti-cancer drugs, for example, with PARP inhibitors to treat cancer cells, ameliorating drug resistance and improving the suppressive efficacy [45].

In preclinical studies, Imetelstat exerted a potent and specific telomerase inhibition in several kinds of tumors including myeloma [46], glioblastoma [47], hepatoma [41]. For the indications of myelofibrosis, myelodysplastic syndromes and thrombocythemia, Imetelstat has active performance and have been proceeded to clinical trails phase III and II respectively. Most of the Imetelstat-related trials registered in ClinicalTrials.gov database areinvolved in the clinical treatment of breast cancer, lung cancer, multiple myeloma and other solid tumors.

In addition, there are several natural small molecules obtained from plant or food sources that have been found to inhibit telomerase activity through modulating telomerase component expression, holoenzyme assembly or blocking enzyme activity. Our lab performed a screening in TERT-P2A-GFP reporter cell line and found a natural product SC as a telomerase inhibitor for multiple cancer types [48]. Epigallocatechin gallate (EGCG), one of the main components of green tea, inhibits telomerase expression and functions depending on epigenetic modification [49]. Further, scientists synthesized MST-199 and MST-312 reagents based on the structure of EGCG, which exhibited stronger telomerase inhibitory efficacy [50]. Quercetin is a natural polyphenol, which can be used for cancer treatment and prevention by down-regulating hTERT expression [51]. Furthermore, quercetin in combination with EGCG could eliminate cancer stem cells [52]. One of the recently synthesized shikonin N-benzyl matrinic acid ester derivatives, PMMB-302, can inhibit telomerase expression and lung cancer cell proliferation [53].

Likewise, siRNAs targeting TERT is an emerging strategy for telomerase inhibition [54]. Using nanoparticles to deliver TERT siRNAs into cells can enhance the cancer suppression efficacy [55, 56]. AAV-mediated gene therapy targeting to telomerase also displays remarkable outcomes in animal models [57]. Resnomics, a biopharmaceutical company from Korea, is pushing forward with its hTERT-targeted adenovirus (RZ-001) therapeutic programs [58], including a preclinical study for glioblastoma and a clinical phase I program for hepatocellular carcinoma. Telomelysin (OBP-301) is a telomerase-specific oncolytic adenovirus, in which an hTERT promoter is used to increase expression of adenovirus early in regions associated with an internal ribosome entry site (IRES) sequence [59]. This construct cause OBP-301 replicates better in tissues transcriptionally expressing high levels of hTERT such as cancer [60]. With the rapid advancement of gene editing technology, more genetic therapeutics may potentiate telomerase as a target of anti-tumor drugs in the future.

Telomeric G-quandruplex is formed by its guanine-rich sequences. G4 ligands can stabilize this motif, resulting in the inability of telomerase to be recruited to elongate telomeres. As a G4 stabilizer, telomestatin was identified to inhibit telomerase activity and trigger telomere shortening in cancer cells [61–63], which was registered for clinical trials of esophageal cancer, melanoma and hepatocellular carcinoma. Also, synthetic oxazolyl-type telomestain derivatives have also been developed and their therapeutic efficacy was validated in preclinical cancer models [64]. Pidnarulex (also known as CX-5461) is undergoing clinical trials for hematologic cancer patients [65].

In the process of tumorigenesis, the transcriptional regulation of hTERT undergoes a pattern of “off to on” changes. While hTERT promoter activation mutations occur in some of cancer cells [66], the mechanism of wild-type hTERT promoter reactivation in a majority of cancer cells is not clear. Akıncılar S.C. and Tergaonkar V. have recently provided a new answer to this question. They identified hTERT interaction region 2 in primary colorectal cancer as an essential chromatin region necessary to regulate the reactivation of wild-type hTERT, facilitating the formation of stable complexes by associated transcriptional regulators to initiate hTERT expression [67]. This action model, in turn, allows us to design more cancer-specific telomerase inhibitors.

**Table 1:** Cancer-specific telomerase inhibitors

Inhibitors	level	Preclinical Model	Mechanism	Efficacy/Progress
BIBR1532	Preclinical	Multiple cancer cells	Specifically block hTR binding site in hTERT	a. Suppressive cancer cell proliferation; b. Enhance chemoradiotherapy
Imetelstat (GRN163L)	Clinical	CDX mice models	Directly bind hTR sequence and competitively inhibits telomere binding	a. Inhibit MM/GBM/HCC in preclinical studies; b. Several cancer related clinical studies are ongoing; c. Active clinical performance in Myefibrosis and Myelodysplastic Syndromes
Sanguinarine chloride (SC)	Preclinical	Multiple cancer cells	Inhibit hTERT/telomerase in a p65-dependent manner	Acute inhibitory effect on telomerase and cancer cell growth
Epigallocatechin gallate (EGCG)	Preclinical	MCF-7 breast cancers cells and HL60 promyelocytic leukemia cells	Decrease hTERT transcription through epigenetic alterations	a. Inhibit cell growth and induce apoptosis in cancers of dissimilar origins; b. Simpler derivatives including MST-312, MST-295, and MST-199 were examined based on the structure of EGCG to exhibit stronger telomerase inhibitory efficacy
Quercetin	Preclinical	Multiple cancer cells	Induce cell cycle arrest, inhibit cell migration, colony formation and downregulate hTERT expression.	Arrest the cell cycle and induce cancer cell apoptosis
PMMB-302	Preclinical	A549 cells	Inhibit the expression of telomerase core proteins, dyskerin and NHP2, and RNA template.	Exhibit the best antiproliferative activity against the A549 cell line
RZ-001	Clinical	liver cancer cells and HCC-bearing mice	Efficiently and specifically target hTERT expression	a. Selectively retard hTERT-positive liver cancers; b. Active clinical performance in HCC and GBM
Telomelysin (OBP-301)	Clinical	Multiple cancer cells	hTERT promoter enables its replication in hTERT transc ription-driven tissues such as cancer	a. Selectively infect and lyse cancer cells; b. Active clinical performance in Hepatocellular carcinoma combination therapy with ICIs will be the most promising therapeutic strategy
Telomestatin	Clinical	Multiple cancer cells	Inhibit the telomerase function through stabilizing G4 at the telomeric repeat	a. Induce cancer cell growth inhibition; b. Registered for clinical trials of esophageal cancer, melanoma and HCC; c. Synthetic oxazole telomestatin derivatives have been developed and verified for their therapeutic efficacies in preclinical cancer models
Pidnarulex (CX-5461)	Clinical	Multiple cancer cells	Inhibit RNA Pol 1, stabilize G4, and inhibit Top2	Undergoing clinical trials for hematologic cancer patients

### Targeted-telomerase immunotherapies in cancers

Human immune system includes both innate and adaptive immunity, which are together responsible for recognizing exotic matters and ultimately protect body from infections. Telomere dysfunction in tissues and organs would accelerate systemic aging, activate inflammatory responses and reduce the innate immunity. Moreover, telomere stress could be sensed by innate immune cells and in turn activate CD8^+^ T cells for tumor killing [68]. Here, however, we are discussing the impact of telomeres or telomerase modulationon tumor immunotherapy, especially on lymphocytes.

Adaptive immune response is a result of substantial and directed expansion of lymphocytes (T and B cells) after invasion by exogenous pathogens. T lymphocytes are categorized into CD4^+^ T cells and CD8^+^ T cells. Of these, CD8^+^ T cells are cytotoxic T cells, which are responsible for killing infected cells or cancer cells.

Telomerase activation is an essential part of T cell immune response, which would be regulated at multiple levels. Although TERT mRNA can be expressed, there is no detectable enzymatic activity in resting T cells [69]. Activated T cells re-express telomerase activity upon antigen presentation in order to satisfy the need of adaptive immune system to complete a rapid expansion [20]. Telomerase could be also activated in synaptic stimulated T cells to develop memory T cells [70]. Furthermore, telomere length is also heterogeneous in different subsets of T cells [71]. Shorter telomeres could be detected in senescent T cells. Overall, the complicated regulation of telomerase plays an important role in the T cell development. Ectopic expression of TERT in human CD4^+^ and CD8^+^ T cells by retrovirus-mediated infection can induce high levels of telomerase activity and maintain telomere length [72, 73], but no proliferation advantage was seen in TERT-transduced CD8^+^ cells [74]. TERT-immortalized T cells were injected back into both rodent [75] and non-human primate [76–78] models, and showed comparable proliferation ability with no loss of antigen recognition, creating a potential new tool for immunotherapy. Interestingly, in cancer cells TERT could activate endogenous retrovirus and induce interferon response which also promote to establish a immunosuppression tumor microenvironment by inhibiting different kinds of T cell populations [79]. Not only that, telomerase is also important for natural killer cells (NK cells) function. Acute myeloid leukemia (AML) is an aggressive malignancy with highly active telomerase, but some evidence suggests that NK cytotoxicity is impaired in AML patients [80]. Dizaji Asl K et al. proposed a double-edged role of BIBR1532 in cancer cell killing and in negative effect on the NK cell activity [81]. It may be worthwhile to try to prove that increasing telomerase activity in hematopoietic progenitor stem cells could contribute to an enhanced anti-tumor immune microenvironment.

A recent study showed that antigen contacted T cells extend telomeres relying on APC cells to deliver telomeric fragments rather than depending on telomerase function [82]. Such ALT-like telomere repair mechanism may in turn provide a novel approach to enhance tumor T-cell therapy.

Though there is no novel pharmaceutical development targeting increased telomerase levels and tumor-killing properties in immune effective cells, substantial evidence in vivo indicated that telomere length and telomerase activity directly impacts immunity against cancer or other diseases. Centenarians with relatively longer telomere length possessed lymphocyte populations which were more sensitive to the immune response [83]. Abnormal telomere shortening usually displays heterogeneous growth and functional abnormalities in multiple organs, which are described synthetically as short telomere syndrome (STS). In pediatric patients with STS, it is more likely to have bone marrow failure and tumors; while in adult patients, the malignant diseases including myelodysplastic syndrome, myelofibrosis, and hepatic and pulmonary fibrosis are more prevalent [84, 85].

Moreover, Armanios M.‘s team has recently found that most STS patients were susceptible only for squamous cell carcinoma of the head and neck, anus or skin, which was associated with immunodeficiency rather than genomic instability, and proved in mice that this susceptibility of STS patients to solid cancers was a result of T-cell exhaustion and impairment of tumor surveillance ecology [86]. Telomere elongation could also promote immunological memory of T cells [82]. Here we summarize several potential telomere elongation modulators in tumor prevention or adjuvant therapy in Table 2.

Telomerase activator 65 (TA-65) is a small molecule telomerase activator extracted from the roots of *Astragalus membranaceus*, which has shown anti-aging and lifespan-prolonging potential [87]. Oral administration of TA-65 were found to extend the average telomere length of leukocytes, increase cytotoxic T cells, as well as help the body to reinforcethe immune system [88]. In addition, cycloastragenol (CAG), as a component of TA65, is also considered to be a potential telomerase activator with anti-aging effects [89]. Studies have shown that CAG, by binding to its target protein cathepsin B, inhibits the lysosomal degradation of major tissue-compatible complex I, promotes the aggregation of MHC-I to the cell membrane and accelerates the presentation of tumor antigens. The combination of CAG and PD-1 antibody can also effectively enhance the killing ability of CD8^+^ T cells in cell-derived xenograft (CDX) mice and colorectal cancer organoids [90]. Remarkably, the activation of telomerase in senescent cells and telomere dysfunctional cells did not increase the risk of oncogenesis. For this point, Blasco M.A.’s lab has already proved the safety of overexpressing TERT in mice models [91, 92].

L-Ascorbic acid (Vitamin C, Vc) can promote tissue regeneration mediated by mesenchymal stem cells via increasing their telomerase activity [93]. It was found that oncoprotein E6 can activate telomerase in HeLa cells, while p53 inhibits telomerase activity by down-regulating the transcription of hTERT gene. Vc was found to reduce telomerase activity in HeLa cells by restoring cell redox potential, inhibiting E6 gene and promoting p53 gene expression [94]. These studies seems controversial on Vc’s role in telomere regulation. Recent studies have suggested that Vc can be used to treat cancer by targeting weaknesses common to many cancer cells, namely redox imbalances, epigenetic reprogramming, and oxygen-sensing regulation [95]. Clinical data show that Vc can provide support for cancer prevention or treatment, and the exact role of Vc in preventing cancer progression remains to be demonstrated by more clinical trials in the future.

**Table 2:** Potential telomere length modulators in tumor prevention

NO	Compounds	Level	Preclinical Model	Mechanism	Efficacy/Progress
1	TA65	Preclinical	Multiple cells	a. Extended the average telomere length of leukocytes b. Increased cytotoxic T cells	Help the body to reinforce the immune system.
2	CAG	Preclinical	Multiple cells	a. accelerates the presentation of tumor antigens b. The combination of CAG and PD-1 antibody can also effectively enhance the killing ability of CD8^+^T cells	Anti-cancer
3	Vc	Preclinical	Multiple cancer cells	a. Reduce telomerase activity in HeLa cells by restoring cell redox potential, inhibiting E6 gene and promoting p53 gene expression b. Target redox imbalances, epigenetic reprogramming, and oxygen-sensing regulation in cancer cells	a. Activate telomerase in HeLa cells b. Treat and prevent cancer

Acting as a tumor antigen itself, overexpression of hTERT can activate the immune response in vivo. Increased intracellular hTERT expression activates human endogenous retroviral genes, and triggers anti-cancer efficacy through a cascade effect that activates virus-associated innate immunity [96]. More interestingly, cancer cells could process endogenous hTERT and then present immunogenic hTERT-derived peptides on the cytotoxic T cells surface via MHC-I and MHC-II, and finally kill cancer cells [97]. hTERT-related vaccines were summarized in Table 3.

**Table 3:** hTERT-related vaccines

NO	Vaccines	Level	Preclinical Model	Mechanism	Efficacy/Progress
1	RIAVAX	Clinical	Multiple cells	a. Induce anti-cancer T cells b. Enter into cancer cells and reduce HSP, HIF-1α and VEGF levels c. Inhibit angiogenesis	Help to treat prostate cancer, pancreas cancer, colorectal cancer, and malignant melanoma
2	UV1	Clinical	Multiple cells	Induce TERT-specific T cell and increase T cell activity	In combination with anti-PD1 therapies for the treatment of advanced malignant melanoma
3	GRNVAC1 and GRNVAC2	Clinical	Multiple cells	Expressed TERT in DCs allow them to present TERT-relative antigens	Help to treat Acute Myelogenous Leukemia
4	GX301	Clinical	Multiple cells	Induces TERT-specific T cell immunity	Help treating prostate and renal cancers
5	Vx-001	Clinical	HLA-A*0201 transgenic mouse	Induce and select TERT-specific T cells	Help treating NSCLC and advanced solid tumors

RIAVAX (also known as GV1001) is an anticancer vaccine derived from the sequence of human telomerase active site [98]. It has been reported that RIAVAX not only penetrates the cytoplasm to reduce HSP levels, but also decreases the expression of HIF-1α and VEGF in cancer cells under hypoxic conditions [99, 100]. RIAVAX is the first vaccine to be tested in non-randomized clinical trials for the treatment of different types of cancer, including advanced pancreatic cancer, non-small cell lung cancer and melanoma [97]. Clinical results demonstrated that RIAVAX significantly prolongs survival in pancreatic cancer patients who respond to CD8^+^ T cells [101].

Yet the general vaccine effect was not high and had no statistical significance on prolonging patient survival in combination therapy. As a result, UV1 was developed as a second-generation telomerase targeted peptide vaccine. UV1 performed well in clinical trials of metastatic hormone-naive prostate cancer [102] and received FDA fast track designation for use in combination with anti-PD1 therapies for the treatment of advanced malignant melanoma.

Expressing hTERT and lysosomal-associated membrane protein 1 (LAMP1) in dendritic cells induces an increased degradation of hTERT by lysosomes into small tumor antigenic peptides, which in turn activate downstream Cytotoxic T Lymphocyte (CTL) responses via antigen presentation. Based on this principle, two dendritic cell- based vaccines, GRNVAC1 and GRNVAC2, have been generated. These vaccines have both presented good tumor suppression and tolerability in clinical trials [101, 103, 104].

GX301 and Vx-001 are also the TERT peptide vaccines in clinical trials for treating cancer. The ingredients of GX301 are TERT_540 − 548_, TERT_611 − 626_, TERT_672 − 686_, and TERT_766 − 780_ peptide segments, as well as two adjuvants named Montanide ISA-51 and Imiquimod. In clinical trials, GX301 can induce specific immune responses in patients with prostate cancer and kidney cancer, extending the progression free survival and overall survival of treatment group [105]. Although multiple immunizations tend to pose a risk of T cell exhaustion, considering that GX301 has not shown serious adverse reactions of patients in multiple dosing regimens, it is advisable to use more doses of vaccine to improve immune response when facing intractable tumors [106]. Vx-001 includes multiple injection cycles of TERT_572_ peptide and TERT_572Y_ peptide. General treatment is to first inoculate with MHC-I restricted TERT_572Y_ peptide to generate TERT specific CD8^+^ T cells; and T cells targeting natural TERT antigens were screened by later vaccination with TERT_572_ peptide [107]. The conducted experiments have shown that Vx-001 can prolong the progression free survival and overall survival of patients with advanced solid tumors, but there are significant individual differences in clinical outcomes within the groups [108–110]. For non-small cell lung cancer (NSCLC), the efficacy of Vx-001 can be predicted by the number of tumor-infiltrating lymphocytes (TIL): Patients with high levels of CD3^+^-infiltrating lymphocytes, CD8^+^-infiltrating lymphocytes, and GZMB^+^- infiltrating lymphocytes are not suitable for immunotherapy with Vx-001 [111].

### Related therapies that target non-canonical roles of telomerase

Targeting telomerase activity seems to be an attractive therapy, however, this approach was failed in clinical trials due to possible side effects on stem cells. Thus an alternative strategy could be considered to target the molecules involved in the non-canonical functions of telomerase components.

TERT can also directly binds to promoters with TCF elements and promote transcription, such as c-Myc and cyclin D1, which are highly expressed in cancer stem cells. MST-312 has been found as a telomerase inhibitor that directly target the TERT and p65 binding interface, which inhibits NF-κB binding to target promoters [112]. MST-312 has been shown to inhibit the proliferation of lung cancer stem cells and reduce tumor volume in mouse model [113, 114]. Treating acute promyelocytic leukemia cells with MST-312 causes the expression of NF-κB-target genes significantly downregulated, without toxicity to PBMC at comparable dosage [114]. Diverse choice of DNA damage response pathways is one of the main causes for radiation/chemotherapy resistance. Liu Y. et al. found that TERT can inhibit non-homologous end-joining and prefer a more accurate homologous recombination pathway for DNA damage repair. Using TERT covalent inhibitor NU-1 to relieve the inhibition of non-homologous end-joining can induce immune infiltration and reduce tumor volume in CDX mice models [115].

Notably, Nagpal N. et al. conducted large-scale screening to identify PAPD5 inhibitors as TERC boosters and restored telomere length in DC cells [116]. Lab of Agarwal S. also found thymidine nucleotide metabolism controls human telomere length [117]. Interestingly, TERC was found to suppress PD-L1 expression by downregulating RNA binding protein HuR. Small compound AS1842856, a Foxo1 activator, inhibited the upregulation of PD-L1 induced by chemotherapy drugs [118]. These findings provide new insights for telomerase-targeting tumor therapy.

## Conclusion

Aging plays a critical role in the development of cancers. Scientists have also tried to synergistically link cell senescence with tumors, such as mitochondrial stress and inflammatory response, hoping to target these senescence-related pathways to prevent or treat cancer. Telomere attrition is one of the recognized biomarkers of senescence. Cancer cells rely on high expression of telomerase activity to escape senescence. Researchers have found many telomere inhibitors, which tend to perform well against cancer cell growth in vitro or in vivo. Tumor immunotherapy is currently one of the most promising approaches to significantly improve survival cycles and even achieve a cure for cancer patients. Either anti-immune cell senescence or re-establishing the immunosuppressive microenvironment of tumors by activating T-cell killing effect through TERT antigenic peptides could effectively intensify therapy. Currently there is no anti-tumor drug approved in the market by targeting telomeres or telomerase, and there are many potential telomerase inhibitors with unsatisfactory prognosis in preclinical and clinical trials. However, with the rapid development of technology, gene editing and epigenetic editing are expected to break through the barriers of small molecule inhibitors and achieve potent telomerase inhibition and tumor suppression.

## Abbreviations

CAR-T: Chimeric Antigen Receptor T-Cell Immunotherapy
TR: Telomerase RNA
hTERT: Human telomerase reverse transcriptase
HIF-1: Hypoxia inducible factor-1
VDR: Vitamin D receptor
IGF-1: Insulin-like growth factor 1
PBMC: Peripheral blood mononuclear cell
AML: Acute myeloid leukemia
TRBD: Telomerase RNA binding domain
hTR: Human Telomerase RNA
PARP: Poly(ADP-ribose) polymerases
EGCG: Epigallocatechin gallate
AAV: Adeno-associated virus
IRES: Internal Ribosome Entry Site
MM: Multiple myeloma
GBM: Glioblastoma multiforme
HCC: Hepatoma carcinoma cell
CDX: Cell-derived xenograft
ICIs: Immune checkpoint inhibitors
mRNA: Messenger RNA
NK: Cell Natural killer cell
APC: Professional antigen presenting cell
STS: Short telomere syndrome
TA-65: Telomerase Activator 65
MHC-I: Major histocompatibility complex class I
PD-1: Programmed cell death protein 1
Vc: Vitamin C / L-Ascorbic acid
MHC-II: Major histocompatibility complex class I
HSP: Heat shock protein
HIF-1α: Hypoxia-inducible factor 1 subunit alpha
VEGF: Vascular endothelial growth factor
FDA: Food and drug administration
LAMP1: Lysosomal-associated membrane protein 1
CTL: Cytotoxic T lymphocyte
NSCLC: Non-small cell lung cancer
TIL: Tumor-infiltrating lymphocytes
GZMB: Granzyme B
CAG: Cycloastragenol
TCF: T-cell factor
PD-L1: Programmed cell death protein1 ligand 1
